# Exploring cultural beliefs and practices associated with weaning of children aged 0-12 months by mothers attending services at Maternal Child Health Clinic Kalisizo Hospital, Uganda

**DOI:** 10.11604/pamj.2019.34.47.16940

**Published:** 2019-09-24

**Authors:** Wakabi Hellen Nandagire, Catherine Atuhaire, Ambirigen Teclar Egeineh, Claude Ngwayu Nkfusai, Joyce Mahlako Tsoka-Gwegweni, Samuel Nambile Cumber

**Affiliations:** 1 Faculty of Medicine, Department of Nursing, Mbarara University of Science and Technology, Mbarara, Uganda; 2 Public Health and Hygiene, Department of Public Health and Hygiene, University of Buea, Buea, Cameroon; 3 Cameroon Baptist Convention Health Services (CBCHS), Yaoundé, Cameroon; 4 Department of Microbiology and Parasitology, Faculty of Science, University of Buea, Buea, Cameroon; 5 Faculty of Health Sciences, University of the Free State, Bloemfontein, South Africa; 6 School of Health Systems and Public Health, Faculty of Health Sciences, University of Pretoria Private Bag X323, Gezina, Pretoria, 0001, South Africa; 7 Section for Epidemiology and Social Medicine, Department of Public Health, Institute of Medicine (EPSO), The Sahlgrenska Academy at University of Gothenburg, Box 414, SE-405 Gothenburg, Sweden

**Keywords:** Cross-sectional, qualitative, cultural beliefs, practices, weaning, children

## Abstract

**Introduction:**

Despite the fact that mothers know the exact age to wean their infants, majority of the mothers do not practice exclusive breastfeeding due to cultural beliefs and practices. The purpose of the study was to explore cultural beliefs and practices associated with weaning children at the Maternal Child Health Clinic Kalisizo Hospital.

**Methods:**

This was a simple qualitative study. Seven in-depth-interviews were conducted among 7 mothers of children within the ages 0-12 months attending post-natal care services using self-generated semi-structured key informant's guide. This took place at the Maternal Child Health Clinic Kalisizo Hospital. Purposive sampling method was used to select mothers for the study. Three themes were generated namely: identification of the different cultural beliefs and practices associated with weaning, how the different cultural beliefs are practiced and the impacts of these cultural beliefs and practices. Data were analysed using thematic analysis.

**Results:**

Although a majority of the mothers knew the recommended age to wean their infants, they did not ignore the ill-informed cultural beliefs, taboos and practices from their elders such as peer pressure, advice and counselling from mother-in laws and teachings from older women who are seen as role models.

**Conclusion:**

Adherence to cultural beliefs, taboos and practices, have a powerful influence on weaning, hence hindering exclusive breast feeding.

## Introduction

Weaning from a breast is a natural inevitable stage in a child's development. It is the switching of an infant's diet from breast milk to other foods and fluids [1]. In most cases, choosing when to wean is a personal decision. It might be influenced by a return to work, the mother's or infant's health, or just a feeling that the time is right. World Health Organisation and United Nations Children Emergency Fund recommends exclusive breastfeeding for the first 6 months [2]. The early introduction of mixed feeding began in the early 19^th^ century western society, where prominent contemporary physicians such as the American Pediatric Society founders-Emmett and Lewis recommended that, weaning begin at around 9-12 months for a child or when the canine teeth had grown because infant mortality rate had increased [3]. Mothers identified cultural factors influencing their decision to mix-feeding their babies, which included pressures by their elders and families to supplement because it was a traditional practice, a belief that breast milk is an incomplete food that does not increase the infant's weight, and the taboo of prohibiting sexual contact during breast feeding [4]. It is believed that herbal fluids, saline, water and honey were used by mothers, mother-in-law's and TBAS as gastric cooling agents during the first 3 days of birth and that it also “cleanses” the new born by promoting elimination of meconium which is believed to be harmful [5]. In Uganda, it has been reported that almost 70% of the children are already on supplementary foods by the sixth month of life although breast feeding continues well into the second year for most children. The traditional weaning foods and weaning practices in Uganda and indeed in many developing countries are reported to be inadequate [6]. In Kalisizo hospital in Uganda, monthly reports show that in September 2017, 70 mothers came for postnatal care but only 29 practiced exclusive breastfeeding for children below six months, 32/50 and 24/45 were partially weaned in the months of October and November respectively. Despite the efforts made on exclusive breastfeeding, cultural beliefs and practices have greatly influenced the weaning of children before the age of 6 months. Consequently, there is little information on cultural beliefs and practices associated with weaning of infants and young children in the country. The aim of this study was to explore these cultural beliefs and practices associated with weaning of children before 6 months in the Maternal Child Health (MCH) Clinic Kalisizo Hospital and their impact on exclusive breastfeeding.

## Methods

Qualitative data collection method was used. Seven in-depth interviews were conducted using key informants' self-generated guides to collect data. The study was carried out at MCH clinic Kalisizo which receives 60-90 clients per day. It provides services like elimination of mother-to-child transmission (EMTCT), immunization, family planning and antenatal care. Kalisizo district hospital has both in patient and out-patient services. Kalisizo hospital lies along the main high way to Mutukula and it is located approximately 34 kilometers by the road south west of Masaka. There are 29 health units in which each refer 15-20 cases in Kalisizo district hospital. Participants of the in-depth interviews were mothers of children aged 0-12 months. These mothers were purposively selected. The inclusion criteria were mothers of child-bearing age with children 0-12 months who were attending services at the Maternal Child Health Clinic Kalisizo Hospital and who were not very sick within the study period.

**Data collection and analysis:** seven in-depth interviews were conducted using self-generated interview guides with semi-structured questions. The potential participants were contacted through a face-to-face interview, the interviewer explained to them and each person was requested to provide an informed consent. Face-to-face semi-structured interviews were done and the process lasted between 30 minutes to 1 hour. The main variable explored was cultural beliefs and practices associated with weaning of children within the ages 0-12 months under the themes: sharing of information, role modelling and unhygienic environment. A tape recorder was used to record the audio. This enabled the researcher not to miss out on any communication by the participants as well as observe the non-verbal clues and data were transcribed word verbatim. The transcribed data were reviewed and coded. Data were then entered into word and saved in a rich text format. Using Ryan and Bernard's techniques for identifying themes [7], transcript data from in-depth interviews were coded and put into themes and sub themes.

## Results

As indicated in Table 1, 4(57.1%) were within the ages 18-30years while 3(42.8%) were between the ages 30-40 years and all were within the catchment area of Kalisizo hospital with an equal representation for the majority of the villages. A total of 4(57.1%) were peasants, 2(28.6%) shop attendants, and only 1(14.3%) was a teacher. Majority 5(71.4%) were married as only 2(28.6%) were not married. Majority of the participants (42.8%) had had a secondary school education.

**Table 1:** Socio-demographic profile of participants

Variable/Description	Frequency	Percent (%)
**Age**		
18-30	4	57.1
31-40	3	42.9
**Occupation**		
Peasant	4	57.1
Teacher	1	14.3
Shop keeper	2	28.6
**Marital status**		
Married	5	71.4
Single mother	2	28.6
**Level of Education**		
Primary	2	28.6
Secondary	3	42.8
Tertiary	2	28.6

**Cultural beliefs and practices associated with weaning of children within the ages 0-12 months:** as presented in Table 2, results reveal that the sharing of information, role modelling and unfriendly user environment were identified as cultural beliefs and practices associated with weaning.

**Table 2:** Themes and categories from in-depth interviews

Theme	Category
Sharing Information	Educative services Peer pressure from friends Advice and counselling from mother-in-laws
Role modelling	Behaviour for mother-in-laws Caring attitude for mother-in-laws Teaching by older women
Unfriendly user environment	Poor Organization Being alienated Unhygienic environment

**Sharing information:** sharing of information which was identified in this study as one of the cultural beliefs and practices influencing weaning involved educative services, peer influence, advice and counselling from mother-in-laws. As for educative services, participants reported that the sharing of information was through the teaching they got from midwives. They mentioned that the midwives told them the exact age to start weaning their babies and what food to give them. According to the results, participants reported that the midwives emphasised that they should give their children any other fluid or food only at six months but because of cultural beliefs, they cannot continue practicing exclusive breastfeeding up to six months. According to a participant, practicing exclusive feeding puts the child at risk of illnesses. *“But according to our culture, we believe that when a child makes two weeks, they have to start bathing the baby into “ekyogero” and as the baby bathes, he should be given some to drink because it is believed that this herb can help the baby manifest if it is having yellow fever. In addition to “ekyogero” they give another herb called “ensugo” for the baby to drink as it is believed that the concoction helps the baby to get satisfied and also aids to prevent some diseases the mother had during pregnancy”.*

Peer pressure, advise and counselling from mother-in-laws were also reported as influential factors of child weaning. A participant stated: *“we also have another belief that every neonate has to get abdominal cramps. So a bitter herb called “omululuza” is also given to the baby starting from 2 weeks. I was also told by the mother-in-law that for proper growth of my twins, I had to look for a breastfeeding cow that was at the same age with my twins (they were 1 month old) and get milk from that cow and start giving to my babies. As I stated giving the milk, I saw a great improvement in the size and weight of my babies. At 3months, I began giving my children pumpkin mashed in milk, but I was told by my mother-in-law not to give “emeere-eyomutaka” root tubers until the child starts talking and to all my children I have not been giving sweat potatoes, Irish potatoes, cassava and anything that grows within the soil”.*

Another participant reported that she was advised by her mother-in-law to use food and other fluids before six months as a substitute for breast milk. *“I did not have breast milk so I was told to boil some water, add glucose and give to the baby. Concerning cultural beliefs, I was told by my mother-in-law not to eat grasshoppers “ensenene” during breastfeeding when the husband has not tested first to prevent the child from “okusoba” that is: the child can get diarrhoea, vomiting and can even die. Also, I believed in “ekyogero”, that is, when a baby is bathed and given the bath water to drink, it treats all kinds of illnesses, the baby will be blessed, prevents “akamiro” tongue tight more especially if “ekifumufumu” is added.*

**Role modelling:** as for role modelling, it involved the behaviour of mother-in-laws, their caring attitude and the teachings by older women. These categories tend to influence weaning. The practice of role modelling emerged from observing the older women especially the way they talked to them. Participants felt that the way these mother-in-laws treated them was similar to their own parents. They regarded the cultural beliefs of their mother-in-laws as very important in the development of a child. *“In addition to the above, when my mother-in-law was taking care of me and my baby, she taught me how to breast feed the baby and to give other supplements like mushroom soup, groundnuts soup and milk to the children. She even demonstrated this while giving the baby. I could not ignore what I was told by her because she was at least experienced in these beliefs associated with weaning”. Another participant reported that she had to follow the elderly attitude as they were regarded as role models: “I began giving my child mushroom soup on the second day because it helps the child to get satisfied, not to feel abdominal cramps and it was given for two weeks since I had to follow the advice and teachings from my elders”.*

**Unfriendly user environment:** results revealed that unfriendly user environment which involves being alienated to family members and unhygienic environment was associated to weaning. Result revealed that practicing exclusive breastfeeding, limits the breastfeeding mother and her child's access to her family members, friends and feeding. This tends to influence weaning the child earlier thus affecting weaning. *“We also have a belief that when a woman is exclusively breastfeeding, any woman in her menstrual period should not be allowed to touch the baby and this was too difficult for them to tell when her menstruation period was. Therefore, this caused her to give her child milk and passion fruit at one month. She was not also allowed to cook or push fire into the charcoal stove when she was exclusively breastfeeding. So to her it was a burden and when she started weaning her baby there was a party welcoming her to the kitchen”.*

Another participant reported that unhygienic environment affects weaning. She reported, *“I encountered the worst experience when I began giving my child other feeds, the baby got diarrhoea and was admitted “era yakoma kuntana” but God helped and the baby got well”. Expressed the participant with a relief smile adding that: “also I suspected that the cause of the diarrhoea was poor hygiene because I had been leaving my child and the younger sisters to feed”* she concluded. Her opinion was also upheld by another participant who opined that unhygienic conditions influence weaning as she states: *“eehh nze” “I got the worst experience in the first two months of breast feeding. When I was staying with my mother-in-law, she was giving my baby “ekyogero” and dry tea to drink claiming that the child cannot get satisfied on breast milk alone, and “ekyogero” could chase away all kinds of illness”*. She added that, *“I should use a spoon made out of black cloth leaf or a piece of banana leaf to scup the fluid and give to the baby”*. Similarly, another participant submitted that: *“I use my hands to feed my baby, I do not have separate utensils for feeding my baby and I was told by my in-laws not to give pumpkin to my baby as it can make the child to become weak”*. These testimonies of these women demonstrate the ignorance rate and the extent to which the cultural beliefs of the people have been harmful to the children who are newly born. It is the hope of this study to recommend better ways of managing the situations.

## Discussion

Despite the fact that midwives emphasized exclusive breastfeeding for 6 months through health education, majority of the mothers gave herbs *(ekyogero)* within the first two weeks of life because they strongly believed that these herbs can make their babies strong, chase away all kinds of illnesses and keeping their babies healthy. The continues existence and persistence of these beliefs are promoted by mothers-in-laws, meanwhile, peer groups have also played a key role in preserving these cultural practices and passing it on to the younger generation in the form of counselling and advise. These mothers submit to these unformed elders and continue to practice this cultural beliefs that have contributed to poor weaning practices. This is not different from what other researchers reported [8].

In addition to “ekyogero”, 2 respondents gave to their babies mushroom soup and glucose water in the first 2 days of their lives because they believed that their babies were not getting satisfied with breast milk alone and had a belief that mushroom soup can promote satisfaction and also prevent an infant from abdominal cramps. The practice of giving infants other fluids within the first two weeks of life, is not only common in the young mothers, but in all mothers irrespective of age and parity. Thus, not in line with the reports done by Imdad A *et al.* [9]. Mothers-in-law demonstrated to lactating mothers how they should practice cultural beliefs, as they could go and gather the herbs, prepare them and give to their babies with a caring attitude, and advised them on how to behave during weaning in respect to the cultural norms and practices. Therefore, a majority of the breastfeeding mothers practiced these cultural beliefs because they never wanted to ignore the teachings and practices from their mother-in-laws.

In this study, a majority of the mothers did not see any problem with cultural beliefs and practices associated with weaning and had no separate utensils for feeding their children. Some 2 participants almost lost their infants when they had just introduced supplementary feeds to their babies due to diarrhoea and also the mother to the exposed infant, had the worst experience when she feared to disclose their status to the mother-in-law who was giving supplementary feeds to her baby. Five (71.4%) of the respondents introduced smashed solid foods to their infants at 3 months of age which is not in line with the recommended weaning age. Therefore, mothers did not meet the World Health Organization and UNICEF standard guideline to breastfeeding children due to traditional, cultural, and social beliefs. This is not different from the researches done in Podor in West Africa [10].

## Conclusion

This study produced a rich description of cultural beliefs and practices associated with weaning children within 0-12 months by their mothers. The data analysis revealed that a majority of the mothers knew the recommended age to wean their infants but adherence to cultural practices and taboos had a powerful influence on weaning practices. Mothers find it impossible to ignore the ill-informed cultural beliefs, taboos and practices from their ill-informed elders, and peer group and mother-in-laws, even if one knew that the practice was unhealthy for their child. Therefore, most of the mothers wanted to please the in laws by adhering to these cultural beliefs and practices. Therefore, majority of the mothers of these children within the ages 0-12 months involved themselves in cultural beliefs and practices associated with weaning hence hindering exclusive breast feeding.

### What is known about this topic

- Cultural beliefs and practices influence weaning only in young mothers;
- Mothers start weaning their infants at 4-6 months.

### What this study adds

- Mothers wean their babies or give supplementary feeds between the ages of 2 days-6 months;
- This study produced a rich description of cultural beliefs and practices associated with weaning children with the ages 0-12months.

## Competing interests

The authors declare no competing interests.
